# Is the meaning of subjective well-being similar in Latin American countries? A cross-cultural measurement invariance study of the WHO-5 well-being index during the COVID-19 pandemic

**DOI:** 10.1186/s40359-023-01149-8

**Published:** 2023-04-06

**Authors:** Tomás Caycho-Rodríguez, Lindsey W. Vilca, Pablo D. Valencia, Carlos Carbajal-León, Mario Reyes-Bossio, Michel White, Claudio Rojas-Jara, Roberto Polanco-Carrasco, Miguel Gallegos, Mauricio Cervigni, Pablo Martino, Diego Alejandro Palacios, Rodrigo Moreta-Herrera, Antonio Samaniego-Pinho, Marlon Elías Lobos-Rivera, Andrés Buschiazzo Figares, Diana Ximena Puerta-Cortés, Ibraín Enrique Corrales-Reyes, Raymundo Calderón, Ilka Franco Ferrari, Carmen Flores-Mendoza

**Affiliations:** 1 Facultad de Psicología, Universidad Científica del Sur, Lima, Peru; 2 South American Center for Education and Research in Public Health, Universidad Norbert Wiener, Lima, Peru; 3 Facultad de Estudios Superiores Iztacala, Universidad Nacional Autónoma de México, Tlanepantla de Baz, State of Mexico Mexico; 4 Facultad de Psicología, Universidad Peruana de Ciencias Aplicadas, Lima, Peru; 5 Facultad de Ciencias Humanas y Educación, Universidad Peruana Unión, Lima, Peru; 6 Departamento de Psicología, Facultad de Ciencias de la Salud, Universidad Católica del Maule, Talca, Chile; 7 Cuadernos de Neuropsicología, Rancagua, Chile; 8 Pontificia Universidade Católica de Minas Gerais, Belo Horizonte, Brazil; 9 Consejo Nacional de Investigaciones Científicas y Técnicas, Buenos Aires, Argentina; 10 Facultad de Psicología, Universidad Nacional de Rosario, Rosario, Argentina; 11 Laboratorio de Investigaciones en Ciencias del Comportamiento (LICIC), Facultad de Psicología, Universidad Nacional de San Luis, San Luis, Argentina; 12 Centro de Desarrollo Humano, Universidad Mariano Gálvez, Guatemala, Guatemala; 13 Escuela de Psicología, Pontificia Universidad Católica del Ecuador, Ambato, Ecuador; 14 Carrera de Psicología, Facultad de Filosofía, Universidad Nacional de Asunción, Asunción, Paraguay; 15 Escuela de Psicología, Facultad de Ciencias Sociales, Universidad Tecnológica de El Salvador, San Salvador, El Salvador; 16 Centro de Estudios Adlerianos, Instituto Alfred Adler Uruguay, Montevideo, Uruguay; 17 Programa de Psicología, Universidad de Ibagué, Ibagué, Colombia; 18 Servicio de Cirugía Maxilofacial, Hospital General Universitario Carlos Manuel de Céspedes, Universidad de Ciencias Médicas de Granma, Bayamo, Granma Cuba; 19 Colegio Estatal de Psicólogos en Intervención de Jalisco A.C., Jalisco, Mexico; 20 Universidade Federal de Minas Gerais, Belo Horizonte, Brazil

**Keywords:** COVID-19, Cross-cultural, Invariance, Well-being, WHO well-being index

## Abstract

**Background:**

There is an urgent need to assess changes in well-being on a multinational scale during the COVID-19 pandemic, thus culturally valid scales must be available.

**Methods:**

With this in mind, this study examined the invariance of the WHO well-being index (WHO-5) among a sample of 5183 people from 12 Latin Americans countries (Argentina, Bolivia, Chile, Colombia, Cuba, Ecuador, El Salvador, Guatemala, Mexico, Paraguay, Peru, and Uruguay).

**Results:**

The results of the present study indicate that the WHO-5 is strictly invariant across samples from different Latin American countries. Furthermore, the results of the IRT analysis indicate that all items of the WHO-5 were highly discriminative and that the difficulty required to respond to each of the five items is ascending. Additionally, the results indicated the presence of moderate and small size differences in subjective well-being among most countries.

**Conclusion:**

The WHO-5 is useful for assessing subjective well-being in 12 Latin American countries during the COVID-19 pandemic, since the differences between scores can be attributed to differences in well-being and not in other characteristics of the scale.

## Introduction

The COVID-19 pandemic has not only created threats to physical health, but it has also had a negative impact on the mental health of the population [1, 2]. The effects of COVID-19 on mental health and well-being are profound and long-lasting [3, 4], extending beyond individuals who have been directly affected by the disease [5]. The COVID-19 pandemic has provoked similar reactions in terms of emotions and concerns at the population level in different countries around the world [6]. Recent studies during the pandemic have reported a decrease in well-being compared to pre-pandemic level, also correlating negatively with the presence of symptoms of anxiety and depression [7]. However, after the peak periods of diagnosed cases and deaths during the first waves of the pandemic, an increase in well-being is observed as a consequence of decreased symptoms of anxiety and depression [7]. Thus, the lower number of deaths during the second wave compared to the first wave of the pandemic and the flexibility of prevention behaviors seem to support the hypothesis that subjective well-being varies as a function of the intensity of the COVID-19 pandemic and associated social constraints [8].

In this context, well-being is an important dimension of perceived quality of life, which can be used as an outcome measure in different populations or as an indicator of the effectiveness of different treatment conditions [9]. Thus, findings on well-being are useful for improving mental health services [10] and guiding governmental decisions in health [11]. Therefore, there is an urgent need to assess changes in well-being on a multinational scale during the COVID-19 pandemic [12]. In this sense, in order to assess possible differences between various cultural groups, culturally valid measurement scales must be available. To do this, scales must be examined with different samples to determine which aspects have universal utility and which are applicable only to certain groups [13]. Without such assessments, it is not possible to be certain of the applicability of the results of cross-cultural studies [14, 15].

One of the most widely used scales to measure well-being in clinical and non-clinical studies is the WHO-5 well-being index (WHO-5) [16], which has been translated into more than 30 languages worldwide [17, 18]. There is no single definition of well-being, since it can be interpreted according to the sociocultural context in which an individual operates [19]. Faced with this, for several years, it has been suggested to develop brief scales that globally assess subjective well-being in a single dimension [20]. The WHO-5 evaluates well-being, understood as the degree of well-being experienced by each person according to a subjective evaluation of their life, which includes a set of cognitive judgments and affective reactions, according to previous experiences, the current state of life and the expectations [21]. The WHO-5 is a brief (less than 1 min), generic rating scale that measures subjective well-being over a 2 weeks period [9] and was developed in response to the need to have a measure that reflects a single dimension with high clinical validity [17]. The WHO-5 is derived from the 10-item version (WHO-10) that included positively worded items to measure subjective well-being and negatively worded items to measure distress [18]. For the case of the WHO-5, only positively worded items were considered that are in accordance with the World Health Organization (WHO) definition of good health, which considers positive well-being as a reflection of mental health [22].

Psychometric evidence for the WHO-5 has been evaluated in different countries, settings, and populations [9, 17, 22–27], resulting in a robust measure of subjective well-being. This has led the WHO-5 to be used in different studies measuring the level of subjective well-being and its relationships with numerous psychological and social variables during the current COVID-19 pandemic [7, 28–30]. However, no studies have been reported using the WHO-5 in multiple Latin American countries during the COVID-19 pandemic. Moreover, a recent study that evaluated the validity evidence of the WHO-5 in 35 countries did not include Latin American countries [9]. This highlights the need to evaluate the usefulness of the WHO-5 as a cross-cultural measure of subjective well-being during the COVID-19 pandemic in the Latin American context.

The comparability of the WHO-5 across countries is an important issue, as different cross-national studies use the scale to assess and compare subjective well-being across countries [9]. However, measurement invariance (MI) is a prerequisite for conducting comparative studies [31]. The absence of MI would not allow for certainty that the presence (or absence) of differences in a construct between different groups can be attributed to real differences in the construct rather than caused by differences in psychometric characteristics of the measurement instrument [32]. Specifically, the absence of MI could be caused by different meanings or understandings of the construct between groups, differences in the degree of social desirability or social norms, different reference points when making self-statements, different responses to extreme items, the presence of items more applicable to one group than another, or the incorrect translation of one or more items [33]. Thus, establishing MI for measures is a growing need [34].

Despite the importance of MI in cross-cultural studies [35], there are still few instruments that assess aspects of mental health and have evidence of cross-cultural MI. Thus, while the WHO-5 was used to compare subjective well-being in 31 European countries before the pandemic [36] and during the pandemic in England, Ireland, New Zealand, and Australia [37], no evidence of MI between countries has been reported in these studies. Even a systematic literature review regarding the WHO-5 has not reported outcome information about MI [17]. Only a recent study [9], but with data collected in 2015, reported the presence of metric invariance but not scalar invariance among 35 European countries. Furthermore, based on Item Response Theory (IRT) models, low levels of differential WHO-5 functioning were observed at medium levels, increasing at more extreme levels.

Cross-country MI and the assessment of certain item characteristics are some of the important psychometric issues that remain unclear about the WHO-5 [38]. Therefore, this study examined the MI of the WHO-5 in samples from 12 different Latin American countries. Additionally, the characteristics and performances of the WHO-5 items were evaluated based on IRT. The use of Classical Test Theory (CTT) models allows for confirming previous psychometric results of the WHO-5, while IRT results improve the understanding of its psychometric properties, since IRT provides information about the difficulty and discrimination capacity of the WHO-5 items, as well as the identification of the items that are the most accurate to measure subjective well-being.

## Method

### Participants

For this study, 5183 individuals from twelve countries (Argentina, Bolivia, Chile, Colombia, Cuba, Ecuador, El Salvador, Guatemala, Mexico, Paraguay, Peru and Uruguay) selected by convenience sampling, with diverse occupational backgrounds, participated. The inclusion criteria to be part of the study were: (1) be of legal age, according to the legislation of each participating country; (2) be a resident of one of the 12 participating countries and; (3) give informed consent. Regarding the inclusion of the 12 countries in the study, a systematic selection was not made, since the participation of as many countries as possible was sought. The inclusion of the countries was the result of a negotiation process based on the potential interest of the researchers from each country in participating and the possibility of meeting the research requirements. The Soper software [39] was used to calculate the minimum number of participants in each country. For this, we considered the number of observed variables (5 items of the WHO-5), the number of latent variables of the model to be evaluated (subjective wellbeing), the anticipated effect size (λ = 0.3), the probability (α = 0.05) and statistical power (1 − β = 0.95). The software recommended a minimum sample size of 100 participants in each country. The average sample size in each country was 432 and ranged from 252 (Bolivia) to 877 (Paraguay). Furthermore, the sample size in each country far exceeded the recommended 5:1 ratio of number of participants to number of items [40].

Only 1509 men (29.11%) participated and the mean age was 33.52 years old (SD = 12.90 years). Participants from Argentina and Guatemala had the highest mean age, while participants living in Cuba and Ecuador had the lowest mean age. Table 1 provides country-specific demographic information.

**Table 1:** Sociodemographic characteristics of participants in the Americas

Socio-demographic data	Argentina (*n* = 325)	Bolivia (*n* = 252)	Chile (*n* = 524)	Colombia (*n* = 372)	Cuba (*n* = 317)	Ecuador (*n* = 451)
Age (M ± SD)	44 ± 16.2	39.3 ± 14.5	36.4 ± 12	30.5 ± 13.1	25.1 ± 7.3	29.1 ± 10.6
Gender, n (%)						
Male	76 (23.4%)	75 (29.8%)	124 (23.7%)	101 (27.2%)	118 (37.2%)	137 (30.4%)
Female	249 (76.6%)	177 (70.2%)	400 (76.3%)	271 (72.8%)	199 (62.8%)	314 (69.6%)
Marital status, n (%)						
Single	144 (44.3%)	125 (49.6%)	257 (49%)	267 (71.8%)	205 (64.7%)	309 (68.5%)
Married	95 (29.2%)	82 (32.5%)	142 (27.1%)	59 (15.9%)	48 (15.1%)	92 (20.4%)
Divorced	30 (9.2%)	31 (12.3%)	41 (7.8%)	13 (3.5%)	13 (4.1%)	32 (7.1%)
Cohabitating	41 (12.6%)	9 (3.6%)	80 (15.3%)	28 (7.5%)	50 (15.8%)	14 (3.1%)
Widow	15 (4.6%)	5 (2%)	4 (.8%)	5 (1.3%)	1 (.3%)	4 (.9%)
Educational level, n (%)						
Incomplete elementary school	0 (0%)	0 (0%)	0 (0%)	0 (0%)	0 (0%)	0 (0%)
Completed elementary school	3 (.9%)	1 (.4%)	1 (.2%)	1 (.3%)	0 (0%)	1 (.1%)
Incomplete high school	5 (1.5%)	5 (2%)	3 (.6%)	8 (2.2%)	4 (.9%)	5 (1.1%)
Completed high school	31 (9.5%)	7 (2.8%)	21 (4%)	64 (17.2%)	5 (1.6%)	72 (16%)
Incomplete technical studies	4 (1.2%)	0 (0%)	8 (1.5%)	7 (1.9%)	1 (.3%)	4 (.9%)
Completed technical studies	32 (9.8%)	15 (6%)	43 (8.2%)	36 (9.7%)	10 (3.2%)	11 (2.4%)
Incomplete university degree	84 (25.8%)	60 (23.8%)	106 (20.2%)	118 (31.7%)	150 (47.3%)	130 (28.8%)
Completed university degree	166 (51.1%)	164 (65.1%)	342 (65.3%)	138 (37.1%)	148 (46.7%)	228 (50.6%)
Employment status, n (%)						
Permanent job	207 (63.7%)	106 (42.1%)	300 (57.3%)	188 (31.7%)	227 (71.6%)	170 (37.7%)
Temporary job	41 (12.6%)	59 (23.4%)	77 (14.7%)	66 (17.7%)	14 (4.4%)	70 (15.5%)
Unemployed	77 (23.7%)	87 (34.5%)	147 (28.1%)	188 (50.5%)	76 (24%)	211 (46.8%)
Area of residence, n (%)						
Urban	309 (95.1%)	243 (96.4%)	452 (86.3%)	344 (92.5%)	278 (87.7%)	338 (74.9%)
Rural	16 (4.9%)	9 (3.6%)	72 (13.7%)	28 (7.5%)	39 (12.3%)	113 (25.1%)
Had COVID-19, n (%)						
Yes	50 (15.4%)	73 (29%)	30 (5.7%)	69 (18.5%)	5 (1.6%)	73 (16.2%)
No	216 (66.5%)	137 (54.4%)	437 (83.4%)	211 (56.7%)	275 (86.8%)	286 (63.4%)
I don’t know, but I think so	19 (5.8%)	28 (11.1%)	15 (2.9%)	53 (14.2%)	9 (2.8%)	52 (11.5%)
I don’t know, but I don’t think so	40 (12.3%)	14 (5.6%)	42 (8%)	39 (10.5%)	28 (8.8%)	40 (8.9%)
Family with COVID-19, n (%)						
Yes	169 (52%)	196 (77.8%)	227 (43.3%)	239 (64.2%)	77 (24.3%)	244 (54.4%)
No	156 (48%)	56 (22.2%)	297 (56.7%)	133 (35.8%)	240 (75.7%)	207 (45.9%)
Friends with COVID-19, n (%)						
Yes	284 (87.4%)	241 (95.6%)	335 (63.9%)	306 (82.3%)	179 (56.5%)	375 (83.1%)
No	41 (12.6%)	11 (4.4)	189 (36.1%)	66 (17.7%)	138 (43.5%)	76 (16.9%)

Socio-demographic data	El Salvador (*n* = 698)	Guatemala (*n* = 324)	Mexico (*n* = 300)	Paraguay (*n* = 877)	Peru (*n* = 360)	Uruguay (*n* = 383)
Age (M ± SD)	29.4 ± 8.9	41.6 ± 12.2	33.6 ± 13.7	31.5 ± 10.9	31.8 ± 10.9	38.9 ± 14.3
Gender, n (%)						
Male	260 (37.2%)	114 (35.2%)	98 (32.7%)	212 (24.2%)	114 (31.7%)	80 (20.9%)
Female	438 (62.8%)	210 (64.8%)	202 (67.3%)	665 (75.8%)	246 (68.3%)	303 (79.1%)
Marital status, n (%)						
Single	507 (72.6%)	126 (28.9%)	168 (56%)	577 (65.8%)	231 (64.2%)	171 (44.6%)
Married	127 (18.2%)	146 (45.1%)	95 (31.7%)	202 (23%)	69 (19.2%)	87 (22.7%)
Divorced	11 (1.6%)	26 (8%)	18 (6%)	25 (2.9%)	17 (4.7%)	43 (11.2%)
Cohabitating	51 (7.3%)	20 (6.2%)	14 (4.7%)	67 (7.6%)	41 (11.4%)	75 (19.6%)
Widow	2 (.3%)	6 (1.9%)	5 (1.7%)	6 (.7%)	2 (.6%)	7 (1.8%)
Educational level, n (%)						
Incomplete elementary school	13 (1.9%)	0 (0%)	0 (0%)	0 (0%)	1 (.1%)	0 (0%)
Completed elementary school	10 (1.4%)	1 (.3%)	0 (0%)	4 (.5%)	0 (0%)	1 (.3%)
Incomplete high school	48 (6.9%)	10 (3.1%)	1 (.3%)	17 (1.9%)	4 (1.1%)	24 (6.3%)
Completed high school	106 (15.2%)	21 (6.5%)	20 (6.7%)	76 (8.7%)	14 (3.9%)	36 (9.4%)
Incomplete technical studies	8 (1.1%)	6 (1.9%)	4 (1.3%)	2 (.2%)	7 (1.9%)	1 (.3%)
Completed technical studies	31 (4.4%)	18 (5.6%)	42 (14%)	20 (2.3%)	21 (5.8%)	38 (9.9%)
Incomplete university degree	279 (40%)	67 (20.7%)	82 (27.3%)	292 (33.3%)	105 (29.2%)	113 (29.5%)
Completed university degree	203 (29.1%)	201 (62%)	151 (50.3%)	466 (53.1%)	208 (57.8%)	170 (44.4%)
Employment status, n (%)						
Permanent job	370 (53%)	223 (68.8%)	142 (47.3%)	487 (55.5%)	150 (41.7%)	268 (70%)
Temporary job	86 (12.3%)	45 (13.9%)	57 (19%)	149 (17%)	77 (21.4%)	24 (6.3%)
Unemployed	242 (34.7%)	56 (17.3%)	101 (33.7%)	241 (27.5%)	133 (36.9%)	91 (23.8%)
Area of residence, n (%)						
Urban	550 (78.8%)	304 (93.8%)	279 (93%)	774 (88.3%)	318 (88.3%)	370 (96.6%)
Rural	148 (21.2%)	20 (6.2%)	21 (7%)	103 (11.7%)	42 (11.7%)	13 (3.4%)
Had COVID-19, n (%)						
Yes	113 (16.2%)	28 (8.6%)	47 (15.7%)	132 (15.1%)	74 (20.6%)	10 (2.6%)
No	348 (49.9%)	256 (79%)	196 (65.3%)	556 (63.4%)	205 (56.9%)	320 (83.6%)
I don't know, but I think so	177 (25.4%)	24 (7.4%)	27 (9%)	94 (10.7%)	53 (14.7%)	5 (1.3%)
I don't know, but I don't think so	60 (8.6%)	16 (4.9%)	30 (10%)	95 (10.8%)	28 (7.8%)	48 (12.5%)
Family with COVID-19, n (%)						
Yes	356 (51%)	190 (58.6%)	208 (69.3%)	465 (53%)	243 (67.5%)	81 (21.1%)
No	342 (49%)	134 (41.4%)	92 (30.7%)	412 (47%)	117 (32.5%)	302 (78.9%)
Friends with COVID-19, n (%)						
Yes	514 (73.6%)	289 (89.2%)	252 (84%)	702 (80%)	310 (86.1%)	152 (39.7%)
No	184 (26.4%)	35 (10.8%)	48 (16%)	175 (20%)	50 (13.9%)	231 (60.3%)

### Instruments

WHO-5 well-being index (WHO-5) [16]. The WHO-5 is a five-item, self-administered measure that assesses general subjective well-being over the past 2 weeks. The Spanish version was used [21]: (1) “I have felt cheerful and in good spirits” [“Me he sentido alegre y de buen ánimo”]; (2) "I have felt calm and relaxed" [“Me he sentido tranquilo(a) y relajado(a)”]; (3) “I have felt active and energetic” [“Me he sentido activo(a) y con energía”]; (4) "I have woken up feeling well and rested" [“Me he levantado sintiéndome bien y descansado(a)”]; (5) “My daily life has had interesting things for me” [“Mi vida diaria ha tenido cosas interesantes para mí”]. People answer the five positively worded items of the WHO-5 on a four-alternative Likert-type scale, from "0 = never" to "3 = always". Thus, the total score ranges from 0 to 15, with higher scores indicating greater subjective well-being.

### Procedure

The study followed all the guidelines for the communication of results of online questionnaires and surveys (CHERRIES) [41] in its adaptation to Spanish [42]. In the 12 countries, data was collected through an online survey, administered using Google Forms© during February 15 through March 25, 2021. The online survey was disseminated via social media and online communication channels, such as Facebook, Instagram, WhatsApp and email. Likewise, the online survey began with a section explaining the objective of the study and the request for informed consent. The study ensured the confidentiality of the participants' information and allowed participants to stop answering the questions at any time.

The evaluations and procedures performed in the study were reviewed by the Institutional Committee for the Protection of Human Subjects in Research (CIPSHI) of the University of Puerto Rico (No. 2223-006), which approved the research protocol to ensure confidentiality of the data, sampling and informed consent. All methods were performed in accordance with the relevant guidelines and regulations. All subjects participated anonymously and voluntarily. In addition, they gave their informed consent online at the beginning of the survey.

### Data analysis

A Confirmatory Factor Analysis (CFA) was performed using the Weighted Least Squares Diagonally Weighted Mean and Variance Corrected Mean (WLSMV) estimator due to the ordinal nature of the items [43]. Model fit was assessed based on the chi-square test (χ2), RMSEA index, SRMR index, CFI and TLI. Regarding the RMSEA and SRMR, values lower than 0.05 indicate an excellent fit; whereas, values between 0.05 and 0.08 express an acceptable fit [44]. Likewise, values greater than 0.95 in the CFI and TLI indices indicate a good fit; while values greater than 0.90 express an acceptable fit [45]. Internal consistency reliability was estimated by calculating Cronbach’s alpha and omega coefficients for categorical variables [46]. Values above 0.70 indicate adequate reliability [47].

The evaluation of MI between countries was carried out based on Multigroup Confirmatory Factor Analysis (MGCFA). The MGCFA consists of a sequence of hierarchical variance models, ranging from configurational invariance, metric invariance, where equality of factor loadings is assumed, scalar invariance, where factor loadings and thresholds are equal, and strict invariance, where in addition to equality of factor loadings and thresholds, equality of residuals is also assumed. The comparison of the different sequences of models was performed with the variation of the chi-square statistic (Δχ2), whose non-significant values (*p* > 0.05) suggest the presence of MI between groups. Likewise, a modeling strategy was used, based on the variations of the CFI index (ΔCFI). A difference of less than < 0.010 would indicate the presence of model MI between different groups [46]. Finally, the variation of RMSEA values (ΔRMSEA) was used, where a difference less than < 0.015 is indicative of MI of the model between groups [48]. Once the MI was tested, composite scores were calculated from the sum of the scale items with the objective of assessing differences in subjective well-being between countries. The magnitude of the differences between countries was calculated using Cohen's d test.

Item and test analysis based on IRT was performed with a 2-parameter Graded Response Model (GRM) [49] (2-PLM) specific for ordinal and polytomous items [50]. Discrimination (a) and difficulty (b) parameters were estimated. The *a* parameter evaluates the slope at which item responses vary as a function of the level of the latent trait; whereas, the *b* parameters evaluate the amount of the latent trait necessary for the item to be responded to. Because the WHO-5 has four response options, there are three b-parameter estimates, i.e., one estimate per threshold. The threshold estimates identify the level of the latent trait at which a person has a 50% chance of scoring equal to or greater than a specific response option. Finally, item information curves (IIC) and test information curves (TIC) were calculated.

Statistical analyses were performed in the RStudio environment for R. Specifically for the CFA, the “lavaan” package was used [51], the MI was performed with the “semTools” package [52] and the “ltm” package was used for the GRM [53].

## Results

### Validity based on internal structure and reliability

Table 2 presents the descriptive statistics of the WHO-5 items (mean, standard deviation, skewness and kurtosis) and the polychoric correlation matrix for each of the countries. Table 3 shows that the unidimensional model of the WHO-5 presents adequate fit indices in all countries, especially in Guatemala (RMSEA = 0.000 [0.000–0.067]; CFI = 1.00; TLI = 1.00) and Mexico (RMSEA = 0.075 [0.027–0.125]; CFI = 0.95; TLI = 0.99). In addition, all items have high factor loadings in all countries.

**Table 2:** Descriptive analysis of the items and polychoric correlation matrix

Country	Items	*M*	*SD*	*g1*	*g2*	Polychoric correlation matrix				
						1	2	3	4	5
Mexico (*n* = 300)	1	1.78	.85	− .13	− .74	1				
	2	1.67	.92	− .01	− .90	.83	1			
	3	1.76	.89	− .05	− .95	.82	.82	1		
	4	1.65	.96	− .03	− .99	.74	.79	.80	1	
	5	1.86	.95	− .34	− .88	.73	.75	.76	.69	1
Guatemala (*n* = 324)	1	2.13	.75	− .35	− .73	1				
	2	1.99	.81	− .41	− .41	.80	1			
	3	2.05	.81	− .41	− .63	.80	.86	1		
	4	1.90	.86	− .20	− .87	.72	.77	.80	1	
	5	2.20	.79	− .53	− .74	.72	.74	.77	.67	1
El Salvador (*n* = 698)	1	1.98	.84	− .29	− .81	1				
	2	1.89	.86	− .21	− .84	.86	1			
	3	1.91	.87	− .20	− .93	.78	.86	1		
	4	1.76	.87	− .09	− .81	.71	.74	.76	1	
	5	1.96	.89	− .37	− .85	.74	.72	.74	.70	1
Cuba (*n* = 317)	1	1.94	.81	− .10	− .99	1				
	2	1.77	.88	− .11	− .84	.84	1			
	3	1.82	.87	− .12	− .88	.83	.77	1		
	4	1.71	.90	− .12	− .80	.67	.68	.80	1	
	5	1.72	.89	− .01	− .91	.71	.62	.74	.71	1
Peru (*n* = 360)	1	1.90	.75	− .07	− .71	1				
	2	1.81	.79	− .09	− .60	.81	1			
	3	1.82	.80	− .06	− .73	.78	.81	1		
	4	1.70	.83	− .01	− .68	.67	.74	.82	1	
	5	1.89	.86	− .27	− .74	.73	.71	.76	.70	1
Bolivia (n = 252)	1	1.82	.74	.05	− .68	1				
	2	1.63	.79	.08	− .67	.75	1			
	3	1.72	.82	− .02	− .67	.76	.69	1		
	4	1.62	.81	.03	− .53	.55	.61	.72	1	
	5	1.84	.85	− .25	− .62	.76	.60	.77	.60	1
Ecuador (*n* = 451)	1	1.88	.77	− .09	− .67	1				
	2	1.81	.78	− .11	− .58	.88	1			
	3	1.82	.80	− .09	− .69	.83	.87	1		
	4	1.70	.79	− .11	− .48	.72	.72	.80	1	
	5	1.86	.87	− .27	− .73	.81	.81	.81	.71	1
Colombia (*n* = 372)	1	2.05	.79	− .34	− .72	1				
	2	1.91	.87	− .25	− .88	.86	1			
	3	1.95	.88	− .32	− .85	.86	.85	1		
	4	1.81	.92	− .25	− .84	.78	.78	.80	1	
	5	2.04	.87	− .45	− .77	.75	.68	.76	.73	1
Chile (*n* = 524)	1	1.73	.72	.15	− .58	1				
	2	1.49	.78	.13	− .39	.82	1			
	3	1.47	.78	.29	− .36	.81	.81	1		
	4	1.32	.84	.18	− .52	.73	.79	.83	1	
	5	1.61	.88	.01	− .74	.77	.68	.73	.70	1
Argentina (*n* = 325)	1	1.76	.72	− .09	− .29	1				
	2	1.70	.82	− .17	− .48	.87	1			
	3	1.76	.83	− .21	− .51	.84	.84	1		
	4	1.69	.86	− .07	− .69	.71	.75	.79	1	
	5	1.81	.82	− .17	− .59	.72	.66	.76	.75	1
Uruguay (*n* = 383)	1	1.92	.73	− .15	− .49	1				
	2	1.84	.76	− .12	− .50	.82	1			
	3	1.83	.81	− 24	− .49	.78	.77	1		
	4	1.75	.81	− .21	− .45	.67	.76	.77	1	
	5	1.92	.80	− 25	− .57	.73	.69	.74	.70	1
Paraguay (*n* = 877)	1	1.95	.79	− .21	− .71	1				
	2	1.85	.83	− .16	− .73	.88	1			
	3	1.86	.83	− .19	− .73	.83	.80	1		
	4	1.70	.88	− .07	− .78	.75	.78	.84	1	
	5	1.92	.91	− .38	− .78	.74	.68	.78	.75	1

*M* Mean, *SD* Standard Deviation, *g1* Skewness, *g2* Kurtosis

**Table 3:** Fit indices, factorial weights and reliability of the unidimensional model in American countries

Model	Country	χ^2^	df	*p*	CFI	TLI	SRMR	RMSEA [90%CI]	Factorial weight					Reliability	
									1	2	3	4	5	α	ω
1	Mexico	13.36	5	.020	.99	.99	.010	.075 [.027–.125]	.89	.91	.92	.86	.82	.95	.92
	Guatemala	3.76	5	.336	1.00	1.00	.007	.000 [.000–.067]	.87	.92	.94	.84	.81	.94	.91
	El Salvador	60.98	5	.000	.99	.99	.020	.127 [.099–.156]	.89	.94	.91	.82	.82	.94	.77
	Cuba	74.59	5	.000	.99	.98	.046	.210 [.169–.253]	.94	.88	.91	.83	.79	.94	.92
	Peru	43.69	5	.000	.99	.99	.023	.147 [.109–.188]	.87	.89	.92	.85	.82	.94	.91
	Bolivia	30.26	5	.000	.99	.98	.040	.142 [.096–.192]	.88	.80	.90	.74	.83	.91	.89
	Ecuador	38.04	5	.000	.99	.99	.018	.121 [.087–.158]	.92	.94	.94	.82	.87	.95	.92
	Colombia	29.22	5	.000	.99	.99	.023	.114 [.076–.156]	.94	.93	.92	.85	.80	.95	.92
	Chile	34.51	5	.000	.99	.99	.024	.106 [.074–.141]	.90	.89	.92	.88	.80	.94	.91
	Argentina	47.67	5	.000	.99	.99	.033	.162 [.122–.206]	.91	.91	.93	.84	.81	.94	.91
	Uruguay	29.32	5	.000	.99	.99	.024	.113 [.075–.154]	.88	.90	.89	.83	.82	.94	.90
	Paraguay	126.79	5	.000	.99	.98	.029	.113 [.142–.192]	.92	.91	.92	.88	.82	.94	.93

χ2, Chi square; df, degrees of freedom; SRMR: Standardized Root Mean Square Residual; TLI, Tucker-Lewis Index; CFI, Comparative Fit Index; RMSEA, Root Mean Square Error of Approximation; α, Cronbach’s Alpha; ω, Omega from Green & Yang, 2009

Based on the CFA results, reliability was estimated for each model in each of the countries. Table 3 reports adequate reliability indices for the WHO-5 unidimensional model in each of the countries evaluated (α ≥ 0.94; ω ≥ 0.77).

### Factorial invariance by country

Table 4 presents the sequences of invariance models proposed for each of the countries participating in the study. It was found that the factor structure of the WHO-5 shows evidence of metric invariance (ΔCFI = 0.01), scalar invariance (ΔCFI = − 0.01) and strict invariance (ΔCFI = 0.00).

**Table 4:** Unidimensional model fit indices and invariance models by country

Unidimensional model	χ^2^	df	*p*	SRMR	TLI	CFI	RMSEA	Δχ^2^	Δdf	*p*	ΔCFI	ΔRMSEA
Total sample	538.24	5	.000	.022	.98	.99	.143	–	–	–	–	–
By country												
Configural	218.34	60	.000	.019	.97	.98	.078	–	–	–	–	–
Metric	207.47	104	.000	.029	.98	.99	.048	51.24	44	.211	.01	− .03
Scalar	337.29	148	.000	.035	.98	.98	.054	71.07	44	.001	− .01	.01
Strict	393.31	203	.000	.043	.98	.98	.047	65.93	55	.149	.00	− .01

χ2, Chi square; df, degrees of freedom; SRMR: standardized root mean square residual; TLI, Tucker-Lewis Index; CFI, Comparative fit index; RMSEA, root mean square error of approximation; Δχ2, Differences in Chi square; Δdf, Differences in degrees of freedom; ΔRMSEA, Change in Root Mean Square Error of Approximation; ΔCFI, Change in Comparative Fix Index

**Fig. 1:**
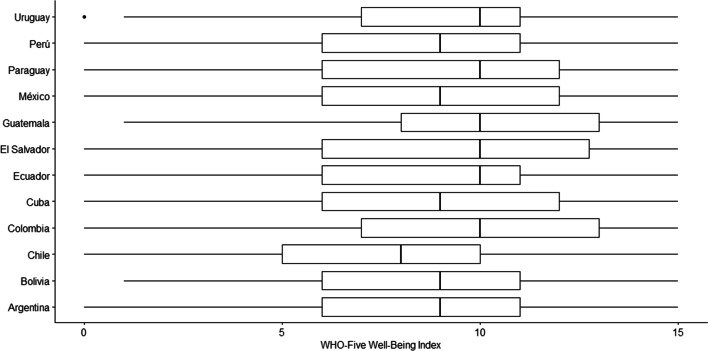
Comparison of scale scores by country

Additionally, Fig. 1 demonstrates graphically the WHO-5 scores in each country. Most of the differences were of moderate and small size. Among the most important results, it was found that Chile presents lower subjective wellbeing scores than Guatemala (*d* = − 0.77), Colombia (*d* = − 0.60) and El Salvador (*d* = − 0.53).

### Item response theory model: graded response model (GRM)

The results of the CFA provide evidence of two important assumptions for IRT, namely the presence of unidimensionality and, consequently, of local independence. In this sense, a 2-PLM GRM was used for polytomous and ordinal items. It was found that all items present parameters greater than 1, which is considered a good discrimination (Zickar et al. 2002). Also, all b parameters increased monotonically. Therefore, a greater presence of the latent trait is necessary for people to respond to the higher response options. All these results are observed in Table 5.

**Table 5:** Discrimination and difficulty parameters for the scale items

Model	Item	a	b_1_	b_2_	b_3_
Unidimensional	WBI1	3.78	− 2.19	− .60	.71
	WBI2	3.90	− 1.81	− .40	.83
	WBI3	4.28	− 1.79	− .41	.74
	WBI4	2.87	− 1.67	− .27	.98
	WBI5	2.61	− 1.93	− .55	.64

a, Discrimination parameters; b, difficulty parameters

**Fig. 2:**
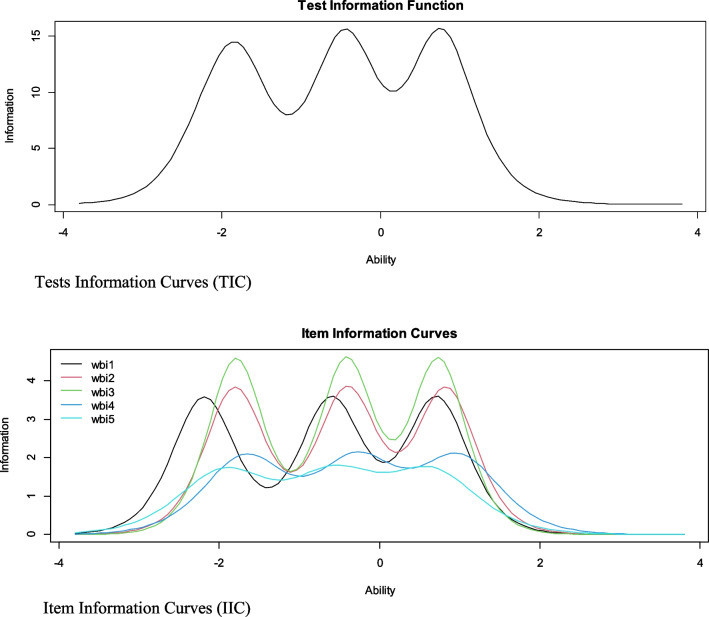
Item and test information curves for the scale

Finally, Fig. 2 shows the IIC and TIC. The IIC indicates that items 3, 2 and 1 are the most accurate in assessing subjective well-being; whereas, the TIC indicates that the WHO-5 as a whole is more reliable in the range of the scale between − 1 and 1.5.

## Discussion

The COVID-19 pandemic has generated public health problems, economic, political and social crises that affect Latin American countries; in addition to having a significant impact on the mental health of the population. For example, it is estimated that, in Latin America, about 231 million people lived in poverty by the end of 2020 [54], in addition to there being a high number of people with severe mental illness who do not have adequate treatment [55]. This leads to the urgent need to have measurement instruments that are useful to identify strategies that promote, prevent, and treat adverse psychological consequences in Latin American countries. Thus, this study aimed to examine the MI of the WHO-5 in samples from 12 different countries.

The results give further support to the evidence of validity and reliability of the WHO-5, demonstrating the presence of solid psychometric properties for the Spanish version applied to Latin American countries. The evaluation of the factor structure of the WHO-5 confirmed the unidimensionality of the scale in the 12 participating countries. This leads to suggest that the Spanish version of the WHO-5 supported the structure of the original scale [17] and that applied in other samples and languages [9]. Similarly, reliability coefficients are very high in each of the countries (α and ω ≥ 0.90), except in the countries of El Salvador (α = 0.94; ω = 0.77) and Bolivia (α = 0.91; ω = 0.89) where they are still acceptable. The unidimensionality of the WHO-5 in the participating countries suggests that all items measure the same construct from a single factor [56]. This would allow for, as with other satisfaction or well-being scales, summing item responses into a total score for use in epidemiological and psychological studies [57]. However, it is important to mention that the RMSEA values, in the factor model, were higher than those recommended in most countries, except Mexico and Guatemala [43, 45]. This is to be expected, since in factorial models with few degrees of freedom, such as the one evaluated in this study made up of five indicators, the RMSEA tends to present a low performance, even if the model is adequately specified [58, 59]. In this regard, it is a mistake to discard factorial models that have high RMSEA values and small degrees of freedom without taking into account other types of information, such as the other fit indices or the factor loadings of the model, which in the case of the current study are very adequate [58].

The results of the IRT analysis indicate that all items of the WHO-5 were highly discriminative, especially item 3 (“I feel active and energetic” [“Me siento activo y enérgico”]). That is, item 3 allows us to adequately distinguish between individuals who have different levels of subjective well-being. This result is consistent with a study conducted in 35 countries where item 3 was also one of the items that allowed for a better and more accurate assessment of people with moderate and high levels of subjective well-being [9]. Item 3 would provide more information on subjective well-being during the COVID-19 pandemic because feeling active and engaging in activities improves quality of life and well-being [60]. Thus, those people who experience subjective well-being may respond more to this item compared to others. Likewise, the difficulty parameter for responding to the items was ascending. This would indicate that a higher level of the latent trait (in this case, subjective well-being) is needed to respond to the higher response categories (high subjective well-being). Finally, the item information curves indicate that the WHO-5 items are more informative at medium or high levels of subjective well-being.

To conduct cross-cultural studies, it is important to conduct MI analysis to assess the possibility that the latent constructs remain the same in different samples from various countries and to generalize the findings to other cultural contexts, as well as to be able to compare levels of subjective well-being across country populations [61, 62]. Overall, the results of the present study indicate that the WHO-5 is invariant at the strict level across samples from different Latin American countries. Specifically, configural invariance was supported, indicating that the unidimensional structure is equivalent across the 12 countries. That is, participants from every country conceptualize subjective wellbeing in a similar way on a single common underlying factor. Furthermore, there is evidence to support metric invariance, where factor loadings were equal across all samples and indicate that people in different countries respond to the items in the same way. The presence of metric invariance is an important prerequisite for meaningful comparisons between different groups [35, 63]. Thus, this finding would allow us to compare regression coefficients and covariance between different groups. Likewise, the presence of scalar invariance indicates that the observed scores are related to the latent scores. In this sense, people who have the same score in the latent construct (subjective wellbeing) would obtain the same score in the observed variable derived from the WHO-5, regardless of whether they belong to one group or another. Scalar invariance is necessary to compare latent means between groups [15]. Finally, strict invariance equated factor loadings, thresholds, and item residuals. The fit of the strict invariance model, observed in this study, would indicate that item measurement errors are the same across countries and that internal consistency is equivalent across the countries assessed [64]. The aforementioned findings support the idea that, when comparing different Latin American countries, it can be assumed that the WHO-5 measures the same psychological construct (subjective wellbeing) in all groups. Therefore, comparisons are valid and differences and/or similarities between countries can be interpreted in a meaningful way [15].

While the main objective of the study was to demonstrate the MI of the WHO-5, we also assessed the differences in scale scores between the participating country samples. For this, composite scores were calculated and not latent variables. Calculating latent variables would have meant choosing a reference group to compare all the other groups [65]. Thus, since it was not possible to identify a single country as a reference group, and considering the importance of assessing the differences between countries, we chose to compare composite measures. The results indicated the presence of moderate and small size differences in subjective well-being among most countries, meaning that people in these countries differed relatively little in their subjective well-being scores during the COVID-19 pandemic. The differences may be partly explained by different orientations towards happiness associated with cultural differences in subjective well-being [66]. This suggests that while the WHO-5 allows for a general assessment of subjective well-being, more in-depth assessments are needed in future studies due to the complexity of subjective well-being [67].

Among the countries that show a greater difference, it was found that Chile presents the lowest subjective wellbeing score, compared to Guatemala (d = − 0.77), Colombia (d = − 0.60) and El Salvador (d = − 0.53). This is explainable, since a previous study showed that Chile is one of the countries with the highest symptoms of dysfunctional anxiety due to COVID-19 in Latin America [68], associated to the significant increase in the number of new infections, due to the false perception of security in the population due to the successful start of the vaccination campaign. This situation could have caused a decrease in the perception of subjective wellbeing of the Chilean population. In general, the differences in means between all countries should be interpreted with caution. More studies are needed to investigate the validity of the WHO-5 in samples that vary, for example, in demographic background. Nevertheless, despite group-level differences, the findings have the potential to add further evidence to the construct validity of the scale.

The current study also has a number of limitations that should be mentioned. First, the study included only a few Latin American countries, mainly from South America (Argentina, Bolivia, Chile, Colombia, Ecuador, Paraguay, Uruguay and Peru), and very few from Central and North America (Cuba, El Salvador, Guatemala and Mexico). Future studies should work with samples from more Latin American countries to obtain more solid conclusions. In addition, the samples in each country were largely comprised of university-educated individuals, who may have certain privileges in terms of socio-economic status and access to health care, which do not necessarily represent the different characteristics in the general populations of each of the countries evaluated. Also, the majority of participants, in all countries, were female. Previous studies have suggested that higher rates of mood, anxiety and stress disorders occur in women [69]. Therefore, gender differences could account for part of the results in this study. However, the difference in the number of men and women in each country did not allow us to examine gender differences in the study. Thus, future studies should investigate the MI of the WHO-5 by sex within countries to assess whether men and women respond to the items differently. Similarly, participants were selected by purposive sampling. All of the above may limit the generalizability of the results and suggest careful interpretation. Additionally, no other measurement instruments were used in this study. Therefore, it was not possible to examine how the WHO-5 is associated with other constructs, which does not provide information about the convergent or divergent validity of the WHO-5. We also did not assess the possible effect of social desirability of responses, which may have been minimized by assuring anonymity in data collection. Also, the cross-sectional design did not allow us to control for cohort effects, nor to assess test–retest reliability and longitudinal MI. Finally, while the total sample (> 5000) may be large [70], the number of participants in some countries may be considered small [71]. Small or moderate sample sizes are common in social science and psychology research [72]. This could generate inadequate conditions for estimating psychometric parameters and the replicability of findings [73]. However, it is important to consider different aspects to assess replicability, such as the large magnitude of factor loadings, and the convergence of methods (CTT and IRT) to assess psychometric properties, which can generate greater confidence in the results obtained. The limitations mentioned here should be considered by future studies to better understand the replicability of the results and to obtain other psychometric evidence needed to complement the substantive WHO-5 research.

Despite these limitations, this study has several strengths. Including several Latin American countries provides more generalizable results with respect to WHO-5 MI than previous studies. Similarly, the findings attempt to fill a gap in the existing literature on the measurement invariance and cross-cultural applicability of the WHO-5 in Latin American countries, thus improving future research in this region. Furthermore, assuring MI is a prerequisite for having an unambiguous interpretation of differences in mean scores and examining the relationships of the WHO-5 with other variables of interest in different settings. Thus, research during the COVID-19 pandemic could incorporate an assessment of subjective well-being as a valid outcome measure in different Latin American countries. In addition, the results may be useful for planning interventions to promote subjective well-being in different Latin American countries. For example, the WHO-5 could be administered to the general population during different periods of the current pandemic or after the pandemic to monitor changes in subjective well-being.

In conclusion, the WHO-5 showed MI in Argentina, Bolivia, Chile, Colombia, Cuba, Ecuador, El Salvador, Guatemala, Mexico, Paraguay, Peru, and Uruguay. This may contribute to the progress of the study of subjective well-being from a cross-cultural perspective. Therefore, this instrument may be useful for assessing subjective well-being in these countries during the COVID-19 pandemic, since the differences between scores in the twelve countries can be attributed to differences in subjective well-being and not to other characteristics of the scale, such as comprehension of the items or familiarity with their response formats. Furthermore, for practical purposes, having a short measure (the WHO-5 has five items) is beneficial for people who have little time to complete longer surveys. Thus, researchers and practitioners can benefit from using the brief and empirically sound WHO-5 to assess subjective well-being in different countries. However, despite the results, future studies on the possible cultural variations in the conceptualization and assessment of subjective well-being could use other more emic approaches based more on the creation of measurement instruments that consider participants' specific cultural perspectives rather than on adaptation or translation.

## Data Availability

The datasets generated during and/or analyzed during the current study are available from the corresponding author on reasonable request.
